# Adenoid cystic carcinoma of the prostate – A rare case of genitourinary malignancy

**DOI:** 10.1016/j.eucr.2022.102025

**Published:** 2022-02-05

**Authors:** Paul Ryan, Caroline Kelly, Sarah Shanahan, Emmet Jordan, John Keane, Padraig Daly

**Affiliations:** a Dept. of Urology, University Hospital Waterford, Dunmore Road, Waterford, X91 ER8E, Ireland; b Department of Medical Oncology, University Hospital Waterford, Dunmore Road, Waterford, X91 ER8E, Ireland

**Keywords:** Prostate, Adenoid cystic carcinoma

## Abstract

A 72 year old was referred to the Urology department with lower urinary tract symptoms (LUTS), an abnormal prostate on digital examination and a serum prostate specific antigen (PSA) level within normal limits.

A flexible cystoscopy revealed no abnormality of the urethra and an obstructive prostate. Magnetic resonance imaging (MRI) revealed a 4.3× 4cm soft tissue mass on the posterior corpus spongiosum encasing the bulbar urethra with tumour abutting the prostate.

Transperineal prostate biopsies confirmed adenoid cystic carcinoma. Cross-sectional imaging arranged for staging, revealed multiple pulmonary metastastis.

The patient is currently being treated with tyrosine kinase inhibitor medication.

## 1 Introduction

Approximately 3900 men are diagnosed with prostate cancer in Ireland each year, which translates to 1 in 7 men being diagnosed with prostate cancer in their lifetime.^1^ The histology involved in prostate cancer varies, but most commonly it is associated with classical adenocarcinoma of the prostate. In rarer cases, different histological types have been reported, such as adenoid cystic carcinoma (ACC).

The prostate is composed of secretory epithelial cells, neuroendocrine cells and basal cells. Prostate adenocarcinoma originates from the secretory epithelial cells, which contain secretory granules and enzymes which stain for prostate specific antigen (PSA), hence the rise in PSA level in cases of benign prostate hypertrophy and prostate adenocarcinoma. ACC however arises from the basal cells of the prostate and therefore is not identifiable on a serum PSA test. It is a malignancy most commonly associated with the salivary glands. ACC of the genitourinary (GU) tract is a rare entity with few cases reported in literature. Most cases of ACC in the GU tract involve the prostate with even fewer cases reporting ACC of the urethra/Cowper's glands.

We herein report the case of a 72 year old gentleman diagnosed with metastatic ACC of the prostate gland.

## 2 Case report

A 72 year old male was referred to the Urology department at our institute with new onset bothersome lower urinary tract symptoms (LUTS), an abnormal feeling prostate on digital examination and a PSA level within normal limits (1.2ng/mL).

**Fig. 1:**
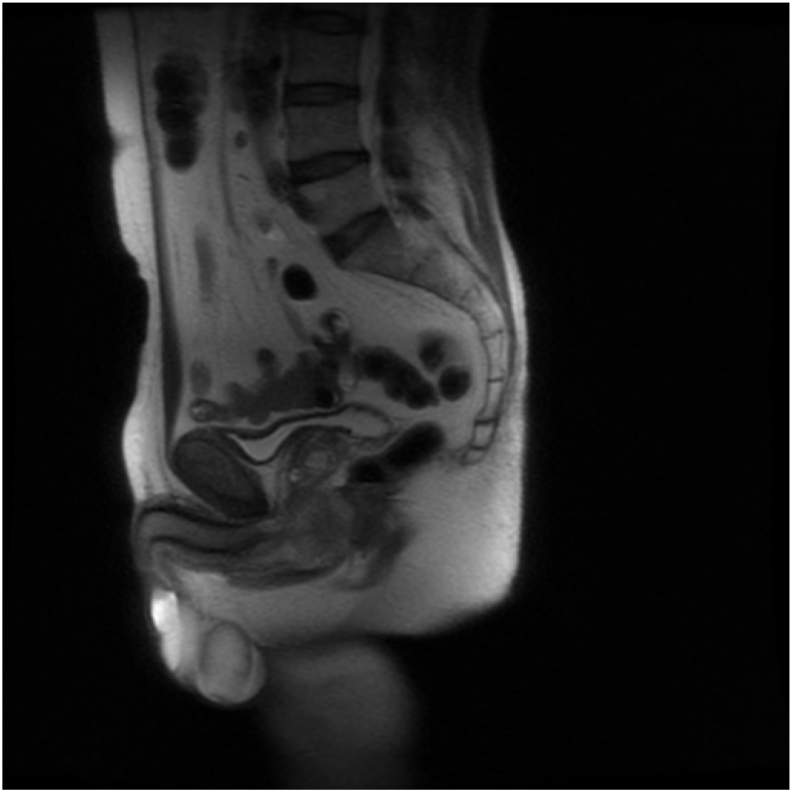
MRI Prostate, sagittal section.

Initial assessment, inclusive of a flexible cystoscopy revealed no mucosal abnormality of the urethra and an obstructive prostate with intravesical protrusion. Magnetic resonance imaging (MRI) revealed a 4.3× 4cm soft tissue mass on the posterior corpus spongiosum encasing the bulbar urethra superiorly with tumour abutting the apex of the prostate (Fig. 1).

The palpable abnormality on digital examination was then targeted with transperineal (TP) biopsies with histology confirming adenoid cystic carcinoma. Cross-sectional imaging was arranged for staging of the disease which revealed multiple pulmonary metastastes. A positron emission tomography (PET) scan was performed to outrule any other primary tumour.

The patient is currently being treated with first-line Sunitinib, a tyrosine kinase inhibitor.^2^

## 3 Discussion

ACC of the prostate is a relatively rare entity, with approximately 100 cases reported in the literature. The proliferation of cells within the basal layer of the prostate often leads to benign prostatic hyperplasia, although rarely it can lead to carcinoma. Billroth first reported adenoid cystic carcinoma in 1859, describing it in the salivary grand with the histological appearance of cribiform, glandular and basaloid patterns.^3^

**Fig. 2:**
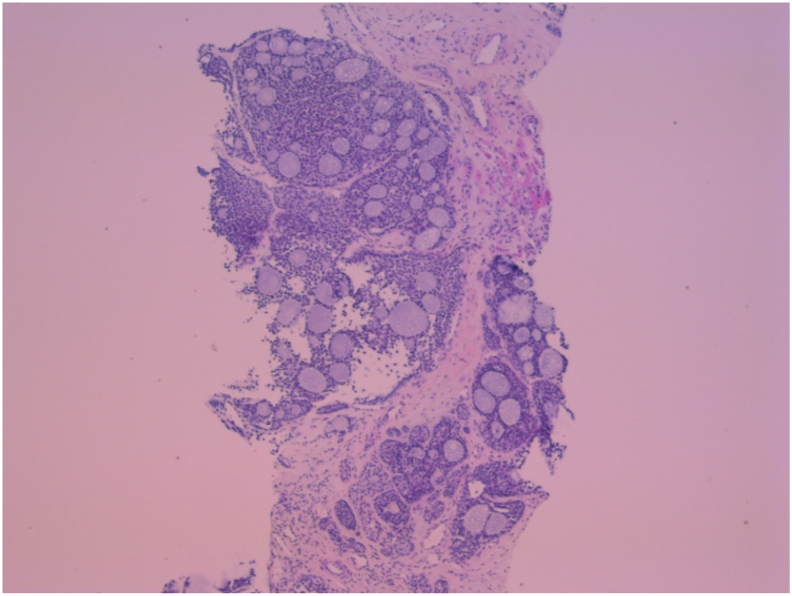
BCC Prostate. Cribiform pattern.

In this case, the histology presented from the TP biopsy is described as cores of fibrous tissue diffusely infiltrated by a basaloid epithelial tumour with a nested solid growth pattern and areas with a cribriform pattern, with associated hyaline globules. The tumour cells are positive for CAM5-2, CKIT, CK7 (luminal cells), BCL2, p16 (large number of cells) and negative for CK20, CDX-2, and TTF-1. Staining for PSA was negative (Fig. 2).

Definitive treatment is hard to identify from the current literature. Some reports suggest surgery, although in a 2018 review of 75 cases by Shibuya et al., they found that in 26 patients who undergo radical prostatectomy, 10 (38%) had disease recurrence.^4^ Prior studies investigating metastatic ACC of the salivary glands found that traditional chemotherapy regimens did not provide a survival advantage.^5^ Chau et al. investigated the value of Sunitinib in recurrent/metastatic ACC of the salivary glands in a single-arm, two stage, phase II trial. Of the 14 patients receiving the treatment, one patient was removed from the trial due to side-effects of the treatment and two patients had disease progression after two cycles of treatment, with a cycle lasting 4 weeks. The remaining eleven patients had stable disease after two cycles, but following a further four cycles, six of these patients had disease progression.^2^

The patient in this case has recently commenced his first phase of treatment with Sunitinib and thus far has not reported any negative side-effects.

## 4 Conclusion

ACC of the prostate is a rare malignancy with symptoms often appearing late in the disease process. Identification of the disease relies almost wholly on a clinical examination guiding a clinician to perform further diagnostic studies, such as prostate biopsies or prostate imaging. Treatment options for localised disease are similar to that used for adenocarcinoma of the prostate, however long-term survival differs greatly. The rarity of the disease coupled with how aggressive it is, makes treatment of metastatic ACC very difficult.
